# Remarks on the Treatment of Incarcerated Herniæ, with Cases

**Published:** 1800-07

**Authors:** M. Ward

**Affiliations:** Surgeon to the Manchester Infirmary


### *Remarks on the Treatment of incarcerated Herniæ, with Cafes: By* M. Ward, *Surgeon to the Manchefter Infirmary.*

HAVING feldom feen the taxis fuccefsful as it is generally conducted, in cafes of incarcerated inguinal and fcrotal hernia, I was induced, feveral years ago, to place the patient in an *inverted pofition** whilft I applied the taxis; and am convinced it poffeffes fome important advantages over the pofitions in common ufe.

The

* By this phrafe is meant, the patient being held by the legs on the fhoulders of an affiftant; his body being allowed to hang down: another affiftant is then to fupport his head and fhoulders, and to incline them a little forwards, fo as to relax the abdominal mufcles.

The following are all the cafes of this kind which have been under my care fince the period alluded to.*

Firft. May 25, 1795, Wm. Bellows was admitted into the Infirmary with a fcrotal hernia, which had been in an incarcerated ftate twelve hours. He complained of great pain, and had vomited frequently.

He was bled copioufly, and had a clyfter given, which produced a trifling evacuation only.

While the warm bath was preparing, an affiftant was directed to fit upon the bed with his back towards the patient, and to grafp his legs (which were put upon his fhoulders) juft above the ancles, and to rife up with him upon his back; another affiftant was then directed to fupport his head and fhoulders, and to incline them a little forwards, principally with a view to relax the abdominal mufcles, but in part to prevent the weight of the body from falling wholly upon the legs. He was held in this pofture ten or fifteen minutes while the taxis was applied, and, by repeating the fame procefs three or four times (allowing him to reft at intervals) the hernia was reduced, without having recourfe to the warm bath.

Aperients and a trufs were directed for him, and he was difcharged cured on the 30th.

Second. January 15, 1796, Wm. Howarth was admitted with an inguinal hernia, which had been incarcerated twenty hours. The moft urgent fymptoms were fingultus, vomiting, and pain.

After fixteen ounces of blood had been taken from his arm, he was placed in the inverted pofition, and the taxis was applied, at intervals, till the warm bath was ready; feveral efforts were alfo made while he was in the bath to reduce the hernia, but were ineffectual. He continued in the bath till he began to grow faint; he was then removed to bed, and, in about an hour after, the reduction was compleated, by again having recourfe to the inverted pofition and the taxis.

Third.

---

* I was not aware until I had arranged my notes on this fubject, that the late Mr. Pott, and Mr. Benj. Bell, had recommended in their Treatifes on Hernia, the pofition which I have called the inverted pofition. The former, after having given directions for applying the taxis, adds, "The pofition of the body, and the difpofition of the lower limbs, may be made very affiftant in this operation, when the difficulty is confiderable; the nearer the pofition approaches to what is commonly called ftanding on the head, the better, as it caufes the whole packet of inteftines to hang, as it were, by the ftrangulated portion, and may thereby difengage it, &c. &c." Pott's Works, (Earle's edition) Vol. II. p. 67, 68.—See alfo Bell's Surgery, Vol. I. p. 284, 3d edition.

Third. March 4, 1796, Job Morton ruptured himſelf by endeavouring to ſtop a reſtive horſe. Several hours had elapſed when he ſent for me.

The hernia was an inguinal one, in a tenſe and painful ſtate. He had vomited ſeveral times. The means made uſe of were venæſection, the inverted poſition, and the taxis, which were happily ſucceſsful.

Fourth. Jan. 20, 1798, Richard Parry, ætat. 40, was admitted with an incarcerated inguinal hernia.

The ſymptoms, mode of treatment, and the reſult, were exactly ſimilar to the firſt caſe. He was diſcharged cured in a few days.

Fifth. Wm. Bellows was admitted the ſecond time, (ſee Caſe 1.) About ſixteen hours had elapſed ſince he found himſelf unable to return the hernia.

He was bled, and the warm bath was ordered; but before it was ready, the complaint was removed, by placing him in the inverted poſition, and applying the taxis.

Sixth. Owing to the following caſe having occurred in the night, it devolved to the care of Mr. Jenkinſon, Houſe Surgeon to the Infirmary, who has favoured me with the following particulars:

" March 17, 1799, Wm. Bellows was brought to the Infirmary at three o'clock, A. M. About ſix hours had elapſed ſince he found himſelf unable to reduce his hernia.

" He complained of great pain, and hiccuped at times.

" I attempted, but in vain, to reduce it by applying the taxis, having previouſly raiſed his hips by placing pillows under them. About a pint of blood was then taken from his arm; a purging clyſter was injected, which brought away a ſtool in about half an hour; a cold ſolution of ſal ammoniac was applied to the part affected, and caſtor oil was ordered internally, which procured him ſeveral evacuations.

" Between ſeven and eight o'clock he was put into the warm bath, and felt eaſier afterwards, though the tumour continued of the ſame ſize, but felt rather ſofter. About two hours after he left the bath, he was held by the legs on the ſhoulders of an aſſiſtant, and the taxis was applied nearly a quarter of an hour, but the hernia was not reduced; he was then laid down in bed, and a ſickneſs came on, and during the continuance of the ſickneſs it went up."

He was diſcharged cured on the 22d, with a new truſs, his old one having been uſeleſs for ſome time.

Seventh. May 13, 1799, James Taylor had experienced the moſt excruciating pain four hours, from an incarcerated inguinal hernia.

The

The inverted poſition was not reſorted to for want of proper aſſiſtants; but the patient's head being lowered, and his hips elevated as much as poſſible, brought him nearly into that poſition; and the hernia was ſpeedily reduced by applying the taxis.

If we admit the propriety of placing the patient, when the taxis is to be applied, in ſuch a poſition as may moſt effectually co-operate with the ſurgeon, the inverted poſition ſeems in every point of view fairly entitled to a preference, not only in the inguinal and ſcrotal, but alſo in the congenital and femoral hernia; and whoever will reflect on the connexion which ſubſiſts between the parts above and below the ſtricture, will be inclined, I think, to draw a ſimilar concluſion.

But this is a matter which does not reſt on theory entirely; the experience I have already had of its utility, enables me to ſay, that more benefit actually takes place from the taxis when the patient is placed in an inverted poſition, and with leſs violence or injury to the diſplaced viſcera, than when any other poſition is made uſe of; an equal degree of preſſure being applied in both caſes.

The alarming ſituation of Wm. Howarth (Caſe 2) induced me both to repeat the taxis oftener, and alſo to apply a greater degree of preſſure before the reſiſtance could be overcome, than I had ever done before; and it is to theſe cauſes, and having availed myſelf of the moſt favourable poſition, that my ſucceſs is to be attributed, not only in this but in the three ſucceeding caſes.

I ſhould be ſorry to be wanting in that reſpect which is due to the opinions of men deſervedly eminent in their profeſſion; but I muſt beg leave to withhold my aſſent from the injunction which would limit the ſurgeon to gentle preſſure in every inſtance. "A little time and pains ſpent in this manner will frequently be attended with ſucceſs, and obtain a return of the part; but if it ſhould not, and the handling of it, (which I muſt repeat ſhould always be gentle) becomes painful and very fatiguing to the patient, we are adviſed to deſiſt for a few hours, and try the effect of other means."*

"But this muſt always be had in view, that any preſſure that is applied muſt be of the moſt gentle kind; for every thing of this nature that creates much pain, is very prejudicial, and ought by all means to be avoided."†

Whoever regulates his practice by the above rules, will, I believe,

* Pott's Works, Vol. II. p. 68.

† Bell's Surgery, Vol. I. p. 287.

believe, rarely fucceed in reducing thofe hernia that are accompanied by a confiderable degree of ftricture.

I truft, however, it will not be imagined I am an advocate for a rude or violent method of applying the taxis; every addition to the preffure applied, fhould certainly be made in a cautious and gradual manner; but at the fame time it will be incumbent upon us to remember, that fhould we fail in reducing the hernia, the patient muft either fubmit to an operation, which often terminates fatally even when performed under the moft favourable circumftances, or to the almoft inevitable alternative of a gangrene taking place, with all its attendant evils.

In the treatment of a complaint fo dangerous in its nature, and fo rapid in its progrefs, that the fate of the unfortunate fufferer is frequently decided in a few hours, it appears highly injudicious to allow a confiderable portion of that precious time (which ought to be appropriated to the ufe of the moft efficacious remedies) to be occupied in applying cold liquids, or ice, to the part affected.*

Where cold topical applications are fo managed as not to divert the attention of the furgeon from meafures of more effential importance, my objections will not apply; but having reafon to believe that great confidence is fometimes repofed in them, and being convinced of their general inutility, I cannot help thinking it would be a happy circumftance were they totally difcarded in the treatment of every fpecies of incarcerated hernia.

In the intervals, when patients of the above defcription are not undergoing the taxis, or ufing the warm bath, might it not promote the object in view (or at leaft retard the progrefs of inflammation) were they to be laid in bed with the head

* Mr. Pott's remarks in his Treatife on Hernia, when cautioning his readers againft employing fomentations, cataplafms, and embrocations, are very forcible, and appear to me not very inapplicable in the prefent inftance. "I know that in this I differ from the majority both of writers and practitioners; but having (as I think) truth on my fide, I do again venture to fay, that I verily believe, that the confidence which has been placed in fuch kind of applications, has deftroyed many more lives than it has faved. A hernia, with painful ftricture, and ftoppage of ftools, is one of thofe cafes in which we can feldom ftand ftill even for a fhort fpace of time; if we do not get forward, we generally go backward; and whatever does no good, if it be at all depended upon, certainly does harm, by occafioning an irretrievable lofs of time: of this kind I take the cataplafm and embrocation to be; while the former is applied, or the latter ufed, no other more powerful means are made ufe of; and though it has the appearance of doing fomething, yet I fear it is little more than fpecious trifling; efpecially if the cafe be at all preffing."
*Works, Vol.* II. *p.* 69, 70.

and ſhoulders lowered, and the hips elevated conſiderably, inſtead of being allowed to lay in the uſual horizontal poſition?

However trivial ſuch a circumſtance may appear, nothing ought to be conſidered beneath notice, which may in any degree tend to promote eaſe, or diminiſh danger.

*Mancheſter, May* 29, 1800.

